# LINC00839 promotes malignancy of liver cancer via binding FMNL2 under hypoxia

**DOI:** 10.1038/s41598-022-16972-z

**Published:** 2022-11-05

**Authors:** Yangyi Xie, Hongsheng Lin, Wei Wei, Yinzhi Kong, Qiaoling Fang, Enran Chen, Jianghua Liu, Mingfen Li

**Affiliations:** 1 Guangxi University of Chinese Medicine, Nanning, China; 2 The First Clinical Faculty of Guangxi, University of Chinese Medicine, Nanning, China; 3 Department of Clinical Laboratory, The First Affiliated Hospital of Guangxi University of Chinese Medicine, Nanning, China; 4 School of General Practice, Guangxi Medical University, Nanning, China; 5 Guangxi Key Laboratory of Chinese Medicine Foundation Research, Guangxi University of Chinese Medicine, Nanning, China

**Keywords:** Oncogenesis, Biomarkers, Molecular medicine

## Abstract

Liver cancer is one of the most common malignant tumors in the world and metastasis is the leading cause of death associated with liver cancer. Hypoxia is a common feature of solid tumors and enhances malignant character of cancer cells. However, the exact mechanisms involved in hypoxia-driven liver cancer progression and metastasis have not been well clarified so far. The aim of this study was to investigate the contribution of long non-coding RNA (lncRNA) in hypoxia promoting liver cancer progression. We screened and revealed LINC00839 as a novel hypoxia-responsive lncRNA in liver cancer. LINC00839 expression was up-regulated in liver cancer tissues and cell lines, and the patients with high LINC00839 expression had shortened overall survival. LINC00839 further overexpressed under hypoxia and promoted liver cancer cell proliferation, migration, and invasion. Mechanistically, LINC00839 bound multiple proteins that were primarily associated with the metabolism and RNA transport, and positively regulated the expression of Formin-like protein 2 (FMNL2). LINC00839 could promote hypoxia-mediated liver cancer progression, suggesting it may be a clinically valuable biomarker and serve as a molecular target for the diagnosis, prognosis, and therapy of liver cancer.

## Introduction

Liver cancer is a major malignancy worldwide ranking as the fourth cause of cancer-related deaths^1^. Hepatocellular carcinoma (HCC) accounts for about 90% of primary liver cancers^2^. Currently, surgical resection and liver transplantation are the most effective therapeutic approaches for patients with early-stage tumors^2^. However, majority of patients with HCC are not eligible for surgery at the time of diagnosis. Besides, the long-term prognosis remains poor due to high occurrence of local invasion and distant metastasis^3^. Better understanding of the critical dependencies and underlying molecular mechanisms in HCC might enable more efficient therapeutic approaches for patients with HCC.

Hypoxia is a common and critical feature of solid tumors, which is attributed to the excessive oxygen consumption due to rapid cell proliferation, and low oxygen supply from insufficient functional blood vessels^4^. Hypoxia microenvironment facilitates cell proliferation, motility, metabolism, drug resistance and stem cell biology^5–7^. In response to hypoxia, the cancer cells alter the transcription of numerous genes and activate multiple oncogenic signaling pathways to coordinate the malignant cell phenotypes^8^. Hypoxia results in the stabilization of hypoxia-induced factor-1 (HIF-1), which helps cancer cells adapt to hypoxic stress by activating gene expression programs that control angiogenesis, glycolytic metabolism, invasion, migration, and erythropoiesis^9,10^. Nevertheless, the molecular details leading to the hypoxic survival advantage of cancer cells have not been fully elucidated.

The human transcriptomic studies demonstrate that the vast majority of genomic sequence is pervasively transcribed into a diverse range of protein-coding RNAs and non-coding RNAs (ncRNAs), of which protein-coding RNAs compose less than 3%^11^. Long non-coding RNA (lncRNA) is defined as a type of transcript with a length of more than 200 nucleotides and no protein coding potential^12^. Increasing evidence has uncovered that lncRNAs are specifically regulated and conserved rather than being the product of transcriptional noise^13^. Their functional mechanisms are diverse, including lncRNAs that act as scaffolds, decoys or signals and can act through genomic targeting, regulation in cis or trans, and antisense interference^14^. Recent discoveries have revealed that lncRNAs drive many important cancer phenotypes through their interactions with other cellular macromolecules including DNA, protein, and RNA^15^. The number of hypoxia-responsive lncRNAs identified in cancer has risen sharply, illustrating the complexity of the hypoxia-induced gene reprogramming and the expanding roles of lncRNAs in hypoxia signaling cascade and responses^16^. It is of great significance to characterize the long non-coding transcriptome involved in hypoxia adaptation for a comprehensive understanding of hypoxia-associated tumor biology.

The high stiffness of the tumor or the surrounding extracellular matrix (ECM) and subsequent loss of mechanical tissue homeostasis are common hallmarks of cell invasion and tumor progression^17,18^. Formins constitute a diverse protein family, which are recognized as potent nucleators of linear actinfilaments that control a large variety of cellular and morphogenetic functions^19^. Formin-like protein 2 (FMNL2), a member of formins, which contributes to the formation of protrusive actin structures such as lamellipodia and filopodia at the leading edge of migrating cells, plays a key role in governing rapid cytoskeletal adaptation to environmental changes and supporting cell efficient adhesion and migration^20,21^.

In the present study, we investigated the contribution of lncRNA in hypoxia promoting liver cancer progression. We identified and verified hypoxia-responsive LINC00839 in liver cancer and systematically investigated the effects of LINC00839 on liver cancer cell proliferation, migration, and invasion under hypoxia. Additionally, mechanistic investigations were performed to explore the potential mechanism of LINC00839 and its connection with FMNL2, which may provide a basis for clinical diagnosis and treatment of liver cancer.

## Materials and methods

### Bioinformatic analysis of RNA-seq and clinical data

The RNA-seq and clinical data of Liver Hepatocellular Carcinoma (LIHC) were downloaded from The Cancer Genome Atlas (TCGA) database (https://cancergenome.nih.gov/). The Ensembl ID was converted to gene symbol, and the expression profile was divided into lncRNA and mRNA using gencode v22 annotation. The differently expressed lncRNAs were identified by DESeq2 package, and log2FoldChange > 1 and padj < 0.001 were set as the threshold for significantly different expression. The co-expression analysis was performed using starBase database^22^. The Gene Ontology (GO) analysis was performed using GO database^23,24^. The Kyoto Encyclopedia of Genes and Genomes (KEGG) pathway enrichment analysis was performed using KEGG database^25–27^. We also labeled tumor samples as “high” or “low” according to whether the expression of FMNL2 was higher or lower than the median value among all samples, and the Kaplan Meier plot was made by R package.

### Cell lines and culture

Liver cancer cell lines Li-7, SNU-387 and SNU-182 were kindly provided by Stem Cell Bank, Chinese Academy of Sciences, and the human liver cell line HL-7702 was obtained from Beyotime (Shanghai, China). All cells were cultured in Roswell Park Memorial Institute (RPMI) 1640 medium supplemented with 10% fetal bovine serum (FBS) and 1% penicillin/streptomycin, and maintained in a humidified atmosphere containing 5% CO_2_ at 37 °C, which was considered as the normoxic condition. To simulate hypoxia, cells were cultured under 1% O_2_ hypoxic condition in a 3-gas incubator.

### Quantitative real-time polymerase chain reaction (qPCR)

Total RNA was extracted from the cells using RNeasy Mini Kit (Qiagen, Germany). Complementary DNA (cDNA) synthesis was performed using the PrimeScript™ RT Master Mix (Takara, Japan). The qPCR was performed using the TB Green® Premix Ex Taq™ II (Takara, Japan) under standard conditions according to the manufacturer’s instructions and conducted with the LightCycler480 System (Roche, Switzerland). The primers were synthesized by Takara (Tokyo, Japan) and the β-actin gene was used as an endogenous control. The data were analysed using the 2^-△△Ct^ method. The primers were listed in Table 1.

**Table 1:** Primer sequences for qPCR.

Gene	Forward (5’–3’)	Reverse (5’–3’)
β-actin	TGGCACCCAGCACAATGAA	CTAAGTCATAGTCCGCCTAGAAGCA
LINC00839	GCTCTCAAGGCTGTTTTCCC	ACATGCACAGAGGGTTGACT
FMNL2	AACCATACAGACGAGCCGAT	TGTGGGCAGTTTCATTCACG

### Cell counting kit-8 (CCK-8) assay

Cell proliferation was determined using CCK-8 (Dojindo, Japan). The 2 × 10^3^ cells per well in 100 μL medium were seeded into 96-well plates and incubated under normoxia or hypoxia for indicated time period, then 10 μL CCK-8 solution was added into each well and incubated at 37 °C for an additional 2 h. Optical density (OD) value at 450 nm was measured.

### Wound healing assay

Cell migration was determined using wound healing assay. The cells were plated to 100% density on 6-well plates and wounded with 1-mL pipette tip each well. The cells were continuously incubated with fresh medium without FBS under normoxia or hypoxia, and wound healing status of each group was observed and photographed at 0 h and 24 h.

### Transwell assay

Cell invasion was determined using transwell assay. Matrigel was dissolved at 4 °C overnight and diluted in medium without FBS at a volume ratio of 1:10, then 100 μL matrigel mixture was added into each tranwell chamber and placed at 37 °C for 2 h to allow the matrigel to solidify. The 1 × 10^5^ cells in 100 μL medium without FBS were added to the upper transwell chamber and 600 μL medium with 10% FBS was added to the lower transwell chamber. After incubation under normoxia or hypoxia for 24 h, cells in the upper chamber were removed and the remaining cells were fixed with 4% paraformaldehyde, stained with 0.1% crystal violet, and photographed.

### Flow cytometry

Cell apoptosis was determined using Annexin V-FITC Apoptosis Detection Kit (Solarbio, China). According to the manufacturer’s instructions, cells were collected and stained with Annexin V-FITC and PI, then analyzed by flow cytometry within 1 h.

### Construction of lentivirus and stable cell lines

Lentivirus that express full-length LINC00839 or LINC00839 shRNA, and their corresponding negative control (NC) lentivirus were constructed by Genechem (Shanghai, China). Lentivirus was infected into SNU-387 cell line respectively for 72 h and 1 μg/mL puromycin was utilized to select stably overexpressed or interfered SNU-387 cell lines.

### Fluorescence in situ hybridization (FISH) assay

The location of LINC00839 in SNU-387 cells was detected using FISH kit (GenePharma, China). Hybridization was carried out utilizing the Cy3 fluorescent dye conjugated probe of β-actin, LINC00839 or NC according to the protocol. The samples were placed under the confocal laser scanning microscope for observation.

### RNA pull down assay

The binding proteins of LINC00839 were collected using RNA pull down Kit (BersinBio, China). Biotinylated RNA probes were incubated with cell protein extract to form RNA–protein complexes. The complexes were separated from other components in the incubated solution through binding to streptavidin-labeled magnetic beads and the proteins were eluted to further analyzed.

### Western blot (WB)

All cells were lysed by RIPA Lysis Buffer (Beyotime, China). The proteins were separated by the sodium dodecyl sulfate polyacrylamide gel electrophoresis (SDS-PAGE) and transferred to polyvinylidene fluoride (PVDF) membranes (Millipore, America). The PVDF membranes were incubated with primary antibodies at 4 °C overnight, then incubated with secondary antibody at room temperature for 1 h, finally incubated with enhanced chemiluminescence (ECL) solution to expose. The antibodies were as followed: β-actin (Cell Signaling Technology, 1:1000), HIF-1α (Cell Signaling Technology, 1:1000), FMNL2 (Abcam, 1:5000).

### RNA immunoprecipitation (RIP) assay

The binding RNA molecules of FMNL2 were collected using RIP Kit (BersinBio, China). The cell extract was incubated with anti-FMNL2 antibody (Santa Cruz Biotechnology, America) or anti-IgG antibody at 4 °C overnight, then protein A/G magnetic beads were added into the samples to incubate at 4 °C for 1 h. Finally the RNA was eluted to subject to qPCR analysis.

### Tissue samples

Tissue microarray chips containing HCC tissues from 64 patients with HCC and adjacent nontumoral liver tissues from 26 patients with HCC were purchased from Shanghai Outdo Biotech (Shanghai, China), and the related clinicopathological and survival information were also provided.

### Ethics declarations

The tissue samples came from National Human Genetic Resources Sharing Service Platform (2005DKA21300). Informed consent was obtained from each patient, and the study protocol was approved by the ethics committee of Shanghai Outdo Biotech Company. All research was performed in accordance with the Declaration of Helsinki.

### Statistical analysis

All statistical analysis was conducted using SPSS 20.0 software. The experimental data was represented by mean ± standard deviation (SD) from at least three independent experiments. The expressions of FMNL2 in tumor and normal samples were compared using Welch’s t test. The survival analysis was performed using log-rank test. The intergroup comparison was carried out using Student’s t test. Differences with a *P* < 0.05 were considered statistically significant.

## Results

### Hypoxia promotes liver cancer cell proliferation, migration, invasion, and the expression of HIF-1α

**Figure 1:**
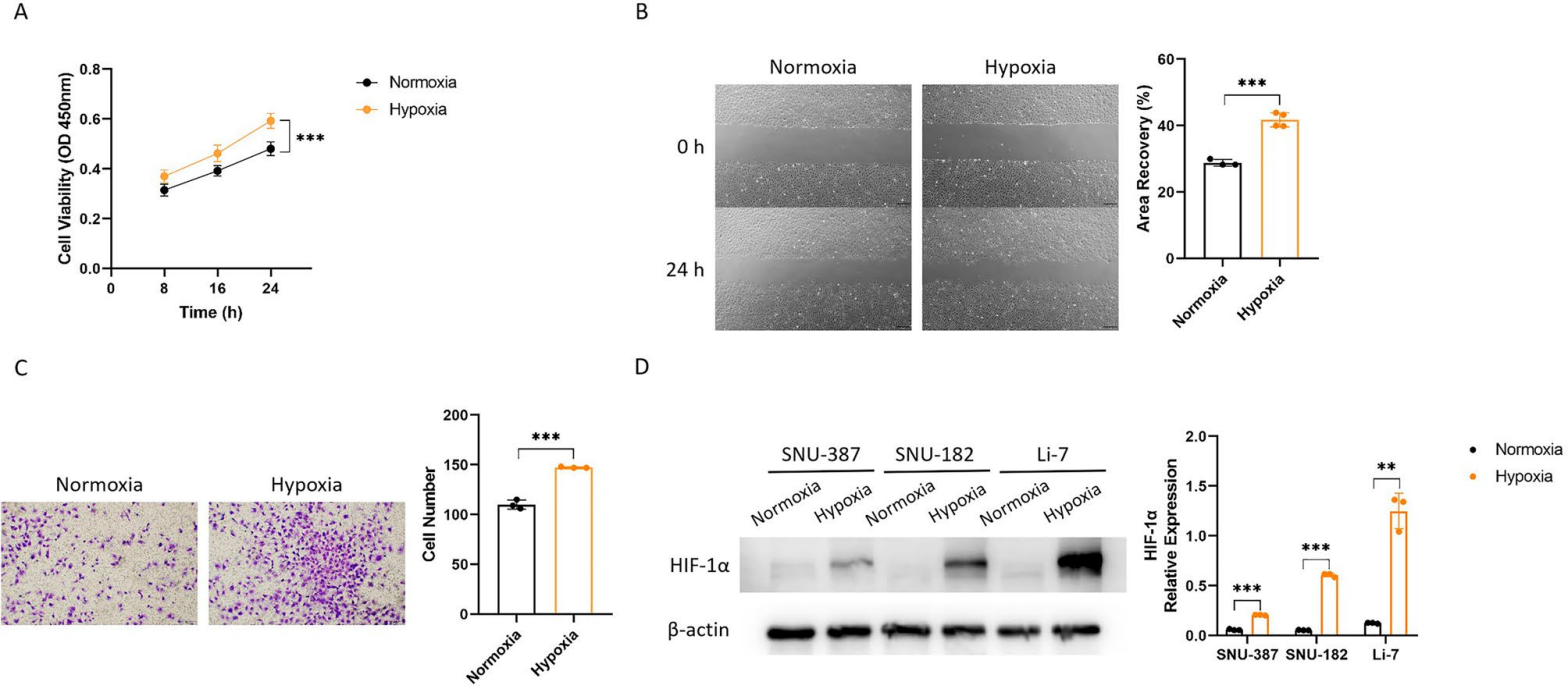
Hypoxia promotes liver cancer cell proliferation, migration, invasion, and the expression of HIF-1α. (**A**) The proliferation ability of liver cancer cells under normoxia and hypoxia by CCK-8 assay (n = 6). (**B**) The migration ability of liver cancer cells under normoxia and hypoxia by wound healing assay. (**C**) The invasion ability of liver cancer cells under normoxia and hypoxia by transwell assay. (**D**) The protein expression levels of HIF-1α in various liver cancer cells under normoxia and hypoxia. The original WB results with markers were shown in Supplementary File 1. The experimental data was represented by mean ± SD from at least three independent experiments. **P < 0.01, ***P < 0.001.

To confirm whether hypoxia promotes the malignant phenotypes of liver cancer cells, we cultured SNU-387 liver cancer cells under normoxic and hypoxic conditions respectively for 24 h. The CCK-8 assay, wound healing assay and transwell assay showed that hypoxia obviously accelerated liver cancer cell proliferation, migration and invasion, compared with that under normoxia (Fig. 1A–C). However, hypoxia did not affect liver cancer cell apoptosis (Supplementary Fig. 1). The WB analysis demonstrated significant up-regulation of HIF-1α in various liver cancer cell lines under hypoxia (Fig. 1D).

### Hypoxia-responsive LINC00839 is selected in liver cancer and validated under hypoxia

**Figure 2:**
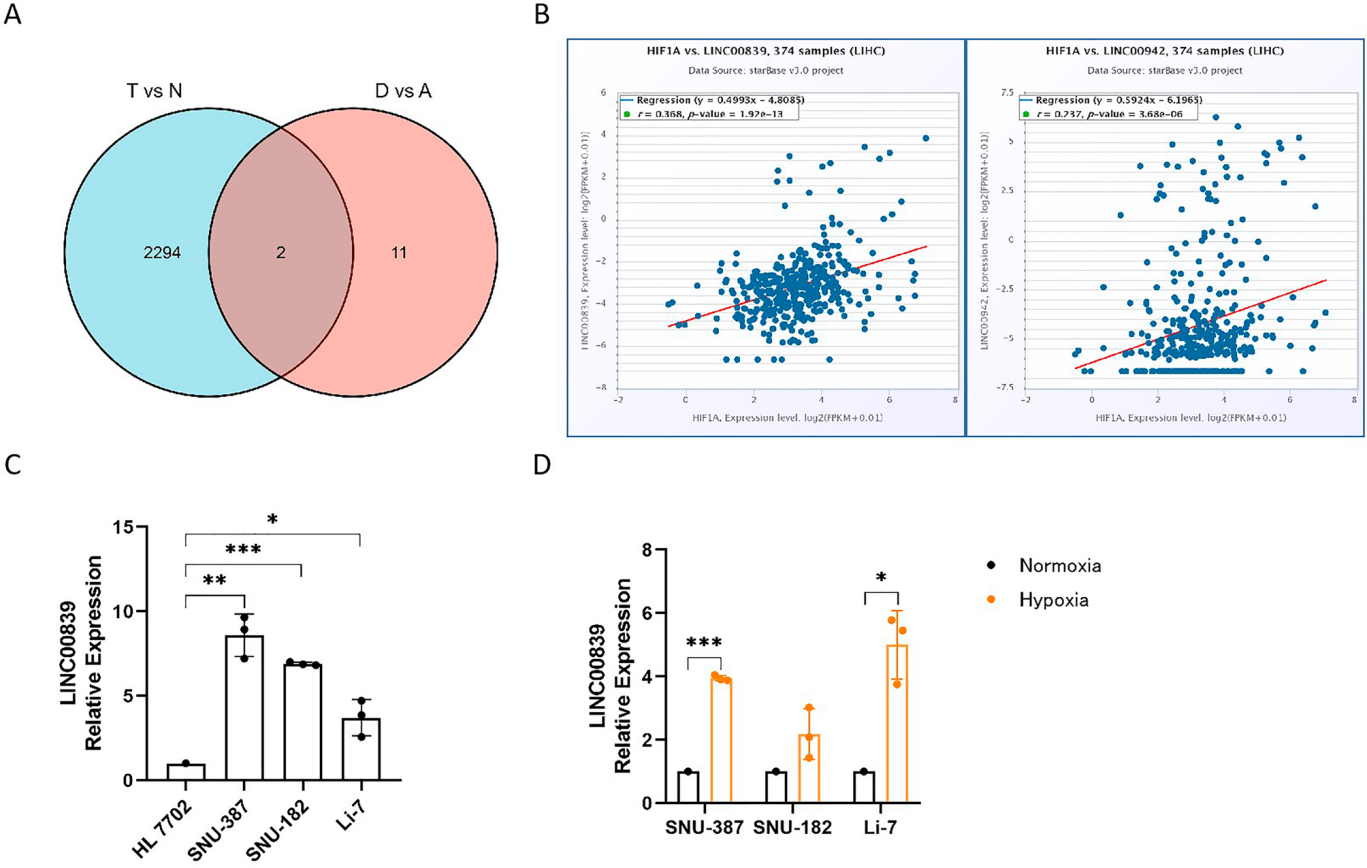
Hypoxia-responsive LINC00839 is selected in liver cancer and validated under hypoxia. (**A**) Venn diagram of differently expressed lncRNAs in two sets. (**B**) The co-expression analysis of LINC00839 and LINC00942 with HIF1A in LIHC. (**C**) The relative expression of LINC00839 in liver cancer cell lines and normal liver cell line. (**D**) The relative expression of LINC00839 in liver cancer cells under normoxia and hypoxia. The experimental data was represented by mean ± SD from at least three independent experiments. *P < 0.05, **P < 0.01, ***P < 0.001.

A total of 14,826 lncRNAs were extracted from TCGA database. We first identified 2296 differently expressed lncRNAs in liver cancer samples (n = 374) and normal samples (n = 50) (T vs. N, log2FoldChange > 1 and padj < 0.001). Then, we unearthed 13 differently expressed lncRNAs between patients with OS.event dead (n = 130) and OS.event alive (n = 235) (D vs. A, log2FoldChange > 1 and padj < 0.001). We screened two lncRNAs overlapped in two sets (Fig. 2A), LINC00839 and LINC00942, that were both up-regulated in liver cancer tissues and correlated with patient unfavorable survival (Table 2). Finally, we selected LINC00839 to carry out further research by co-expression analysis with HIF1A in LIHC (n = 374), whose coefficient-r was higher (Fig. 2B).

**Table 2:** Two lncRNAs overlapped in two sets.

gene_name	log2FoldChange (T vs. N)	padj (T vs. N)	log2FoldChange (D vs. A)	padj (D vs. A)
LINC00839	1.238584	3.081313e-06	1.194333	1.742271e-08
LINC00942	6.142865	1.113416e-24	2.087280	5.554502e-05

T versus N means different expression between liver cancer samples (n = 374) and normal samples (n = 50). D versus A means different expression between patients with OS.event dead (n = 130) and OS.event alive (n = 235). The log2FoldChange > 1 and padj < 0.001 were set as the threshold for significantly different expression.

We examined the expression of LINC00839 in liver cancer cell lines and normal liver cell line using qPCR to validate its expression pattern. The results showed that its expression levels in liver cancer cell lines were approximately three to eightfold higher than that in normal liver cell line (Fig. 2C). What's more, our data demonstrated that LINC00839 tended to increase in liver cancer cells under hypoxia compared with normoxia, with significant differences in SNU-387 and Li-7 liver cancer cells (Fig. 2D), which suggested that LINC00839 may be involved in the promoting effect of hypoxia on liver cancer progression.

### LINC00839 facilitates liver cancer cell proliferation, migration, and invasion under hypoxia

**Figure 3:**
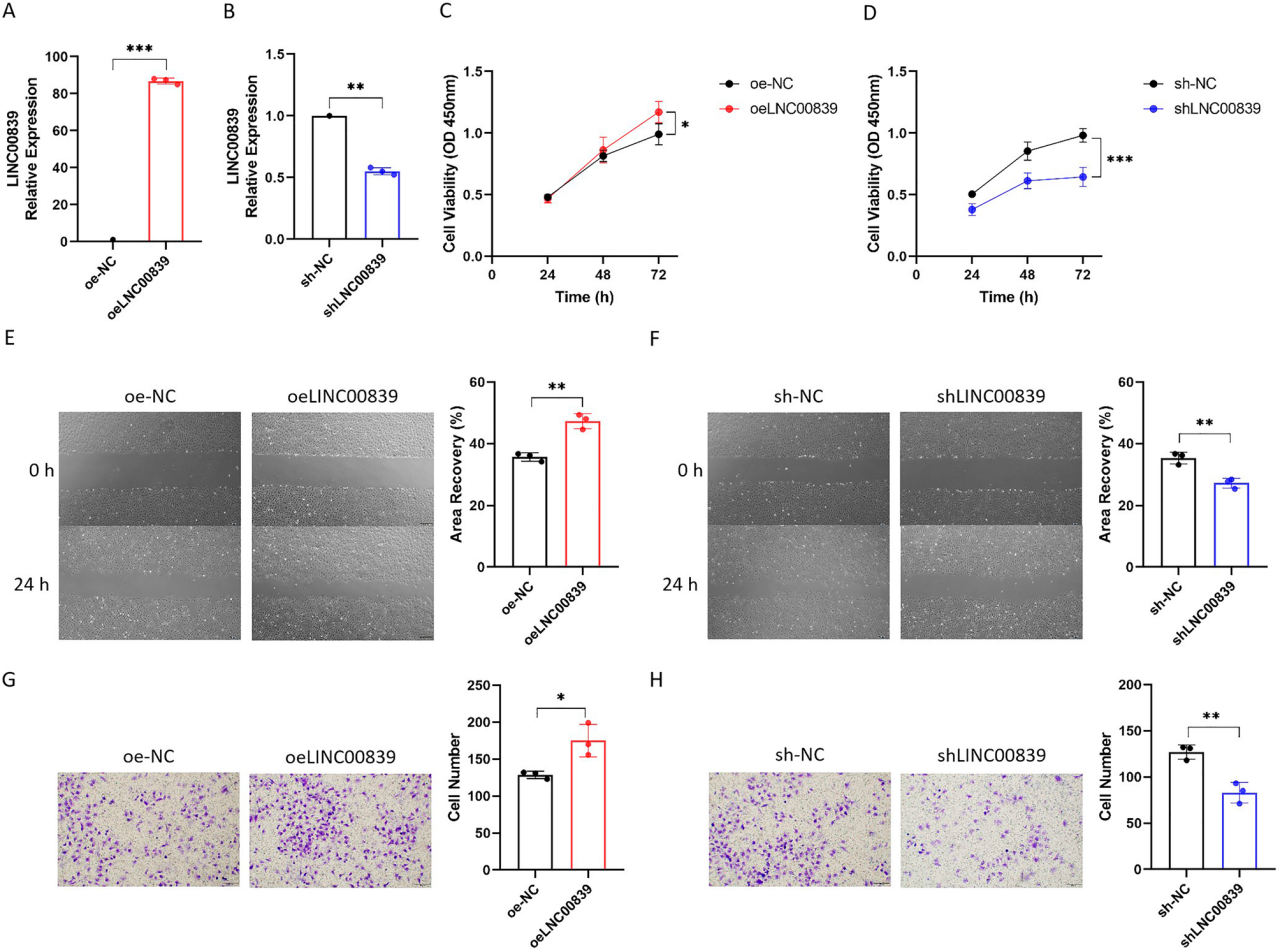
LINC00839 facilitates liver cancer cell proliferation, migration, and invasion under hypoxia. (**A**) The relative expression of LINC00839 in liver cancer cells after infected with LINC00839-overexpressing lentivirus (oeLINC00839) and overexpressing NC lentivirus (oe-NC) under hypoxia. (**B**) The relative expression of LINC00839 in liver cancer cells after infected with LINC00839 shRNA lentivirus (shLINC00839) and shRNA NC lentivirus (sh-NC) under hypoxia. (**C**,**E**,**G**) The proliferation, migration, and invasion ability in the oeLINC00839 group and oe-NC group under hypoxia. (**D**,**F**,**H**) The proliferation, migration, and invasion ability in the shLINC00839 group and sh-NC group under hypoxia. The red bar represented oeLINC00839 group, the blue bar represented shLINC00839 group, and the black bar represented oe-NC and sh-NC group. The experimental data was represented by mean ± SD from at least three independent experiments. *P < 0.05, **P < 0.01, ***P < 0.001.

To estimate the function of LINC00839 on the malignant phenotypes of liver cancer cells under hypoxia, we constructed LINC00839-overexpressing lentivirus and LINC00839 shRNA lentivirus, and then established stably overexpressed or interfered SNU-387 cell lines. We cultured cells from each group under hypoxia for 24 h and verified the expression of LINC00839 (Fig. 3A,B). First, the CCK-8 assay showed that when LINC00839 was overexpressed, the proliferation ability of SNU-387 cells was significantly increased under hypoxia, while interference of LINC00839 markedly decreased the proliferation ability of SNU-387 cells (Fig. 3C,D). Next, we performed wound healing assay and transwell assay to assess the migration and invasion abilities of SNU-387 cells under hypoxia. The results demonstrated that exogenous expression of LINC00839 dramatically facilitated migration and invasion in SNU-387 cells, in contrast, LINC00839 interference significantly inhibited the migration and invasion in SNU-387 cells (Fig. 3E–H).

### LINC00839 is located in both the nucleus and cytoplasm of liver cancer cells

**Figure 4:**
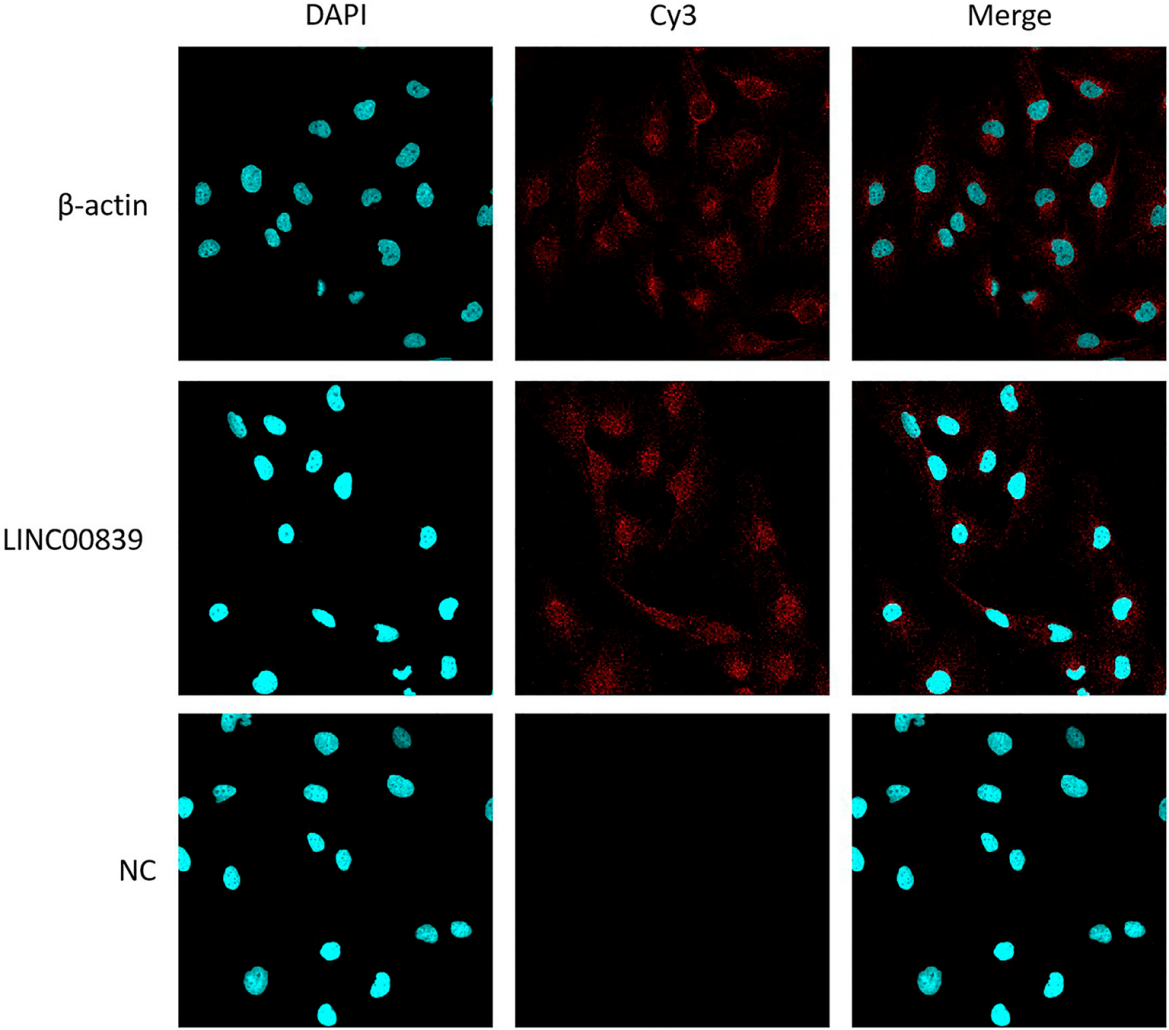
LINC00839 is located in both the nucleus and cytoplasm of liver cancer cells. The unrelated sequence to human gene was used as NC.

To further investigate the oncogenic function of LINC00839 in liver cancer, we detected the location of LINC00839 in SNU-387 cells. The FISH assay displayed that comparing with β-actin, LINC00839 was located in both the nucleus and cytoplasm (Fig. 4).

### Functional enrichment analysis of the binding proteins of LINC00839

**Figure 5:**
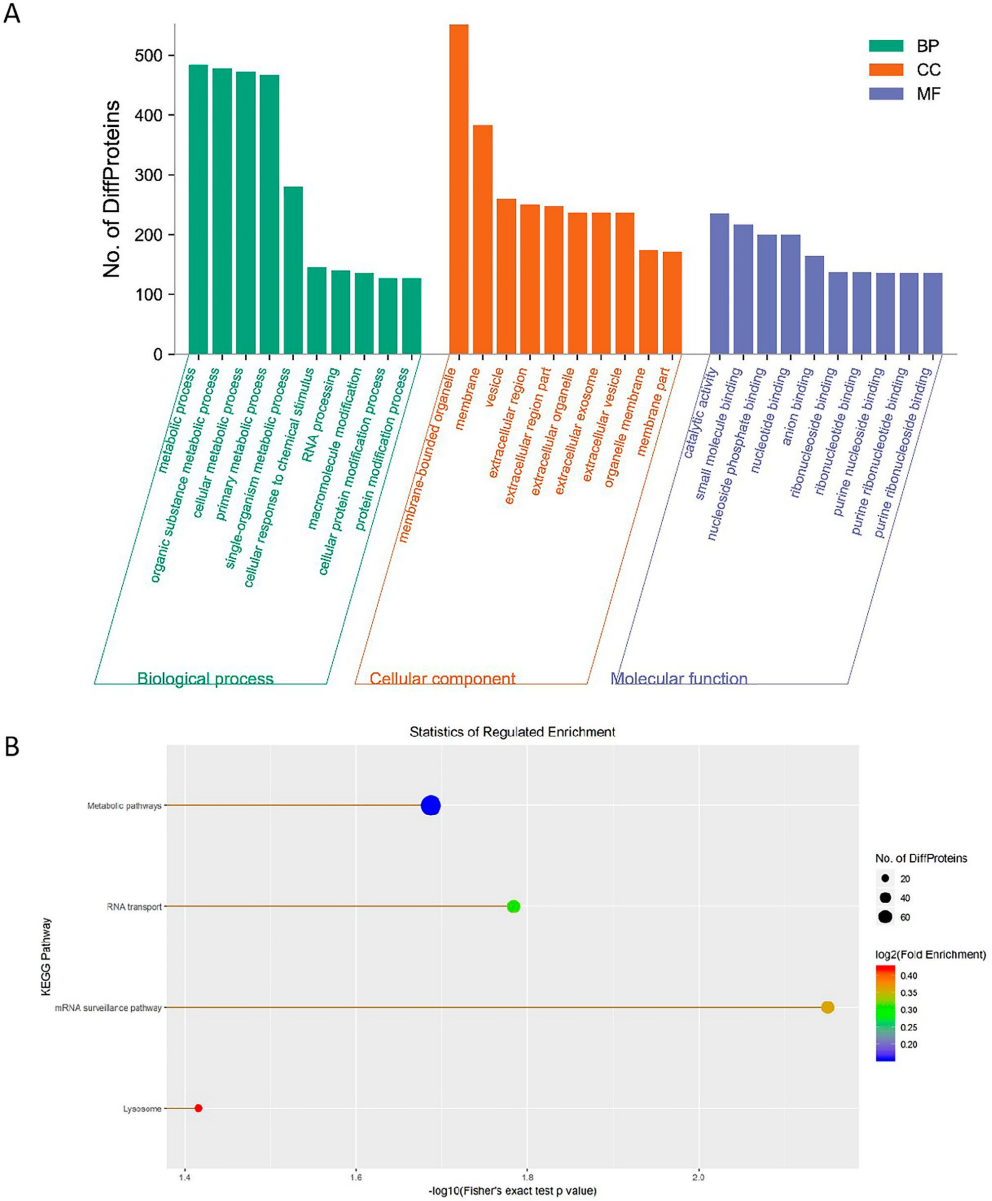
Functional enrichment analysis of the binding proteins of LINC00839. (**A**) Bar plot of GO enrichment in biological process (BP), cellular component (CC) and molecular function (MF) terms. (**B**) Bar plot of KEGG pathway enrichment terms.

We then performed RNA pull down assay using a biotin-labeled LINC00839 probe to identify potential binding proteins of LINC00839 in liver cancer. Mass spectrometry (MS) was used to analyzed the proteins pulled down by LINC00839 and the results were available via ProteomeXchange with identifier PXD034526. In order to predict the potential biological function and connection of the binding proteins of LINC00839, GO and KEGG pathway enrichment analysis were performed. According to the distribution of the binding proteins of LINC00839 in the GO enrichment analysis, the number of proteins was statistically analyzed with significant enrichment of each GO term to clarify protein function in biological process (BP), cellular component (CC) and molecular function (MF). It demonstrated that the proteins participated in various BP terms, especially metabolic process (Fig. 5A). And the most enriched CC term was related to membrane-bounded organelle while the most enriched MF terms were related to catalytic activity and small molecule binding (Fig. 5A). The KEGG pathway enrichment analysis showed that the related pathways were mainly involved in metabolic pathways and RNA transport (Fig. 5B).

### LINC00839 up-regulates the protein expression level of FMNL2

**Figure 6:**
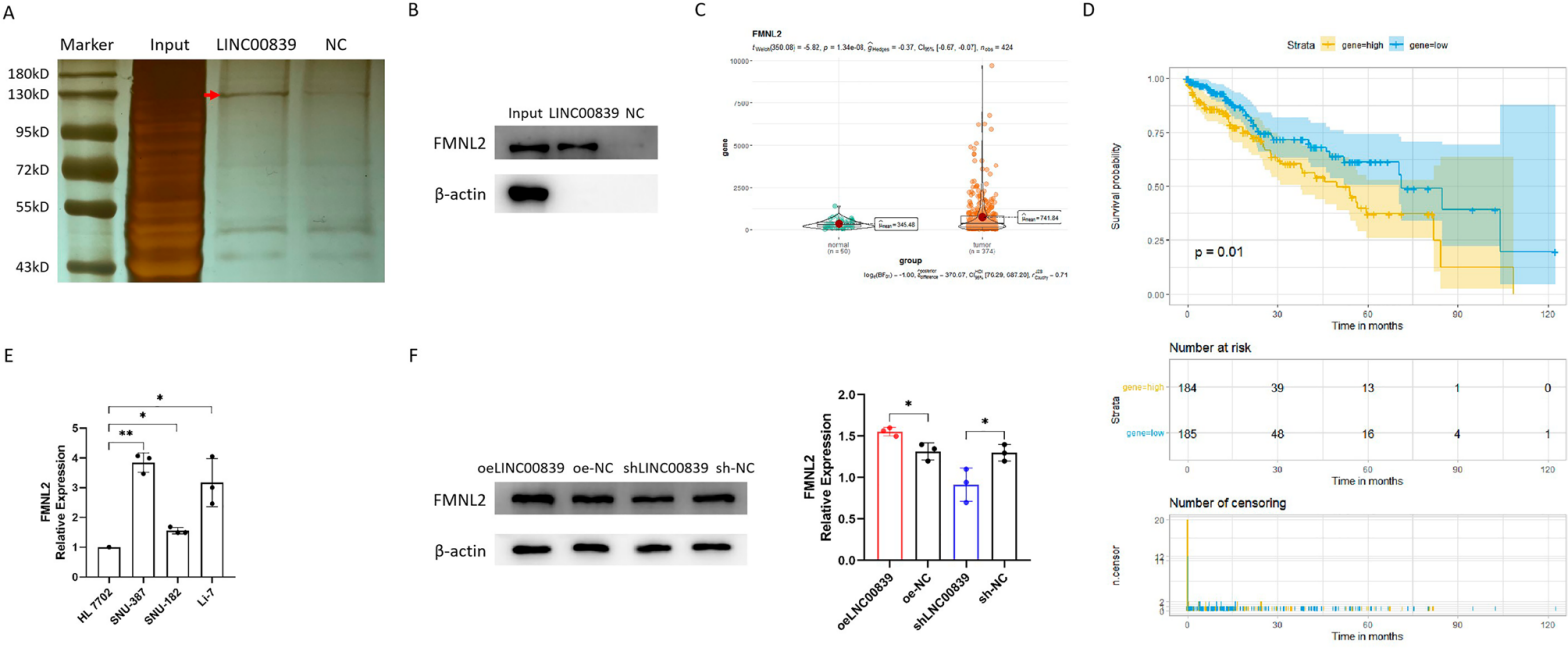
LINC00839 up-regulates the protein expression level of FMNL2. (**A**) The protein samples were separated via SDS-PAGE followed by silver staining. The lacZ gene was used as NC. A specific band was identified in the LINC00839 group and was marked with an arrow. (**B**) The protein samples were examined using WB. (**C**) The expression of FMNL2 in liver cancer samples and normal samples. (**D**) The correlation between FMNL2 levels and patient OS. (**E**) The relative expression of FMNL2 in liver cancer cell lines and normal liver cell line. (**F**) The protein expression levels of FMNL2 in the oeLINC00839 group, oe-NC group, shLINC00839 group, and sh-NC group. The original WB results with markers were shown in Supplementary File 1. The experimental data was represented by mean ± SD from at least three independent experiments. *P < 0.05, **P < 0.01.

The protein samples pulled down by LINC00839 probe and NC probe were separated via SDS-PAGE followed by silver staining. Compared with NC group, LINC00839 pulled down more proteins, especially a specific band around 130 kD (Fig. 6A). According to the MS results, we speculated it was FMNL2 that specifically bound to LINC00839, and used WB to examine. The results showed that FMNL2 was identified in the protein complexes pulled down by LINC00839 but not in those pulled down by NC probe (Fig. 6B). We compared the expression levels of FMNL2 in liver cancer and normal samples and its prognostic significance using the RNA-seq and clinical data from TCGA database. It was found that FMNL2 was highly expressed in liver cancer and elevated expression level of FMNL2 was associated with worse overall survival (OS) of liver cancer patients (Fig. 6C,D). Consistently, it was also observed that FMNL2 was significantly up-regulated in liver cancer cell lines compared with normal liver cell line (Fig. 6E). Besides, the protein expression levels of FMNL2 were determined and compared between the LINC00839 stably overexpressed and interfered cell lines. The results demonstrated a significantly increased FMNL2 expression in the oeLINC00839 group and a decline in FMNL2 in the shLINC00839 group (Fig. 6F). In addition, RIP assay was employed to enrich RNA molecules that bind to FMNL2, followed by examination of the enrichment of LINC00839 in each group through qPCR. However, the results showed that anti-FMNL2 antibody couldn’t enrich LINC00839, compared with IgG group (Supplementary Fig. 2).

### LINC00839 is up-regulated in HCC tissues and correlates with patient clinicopathological features

**Figure 7:**
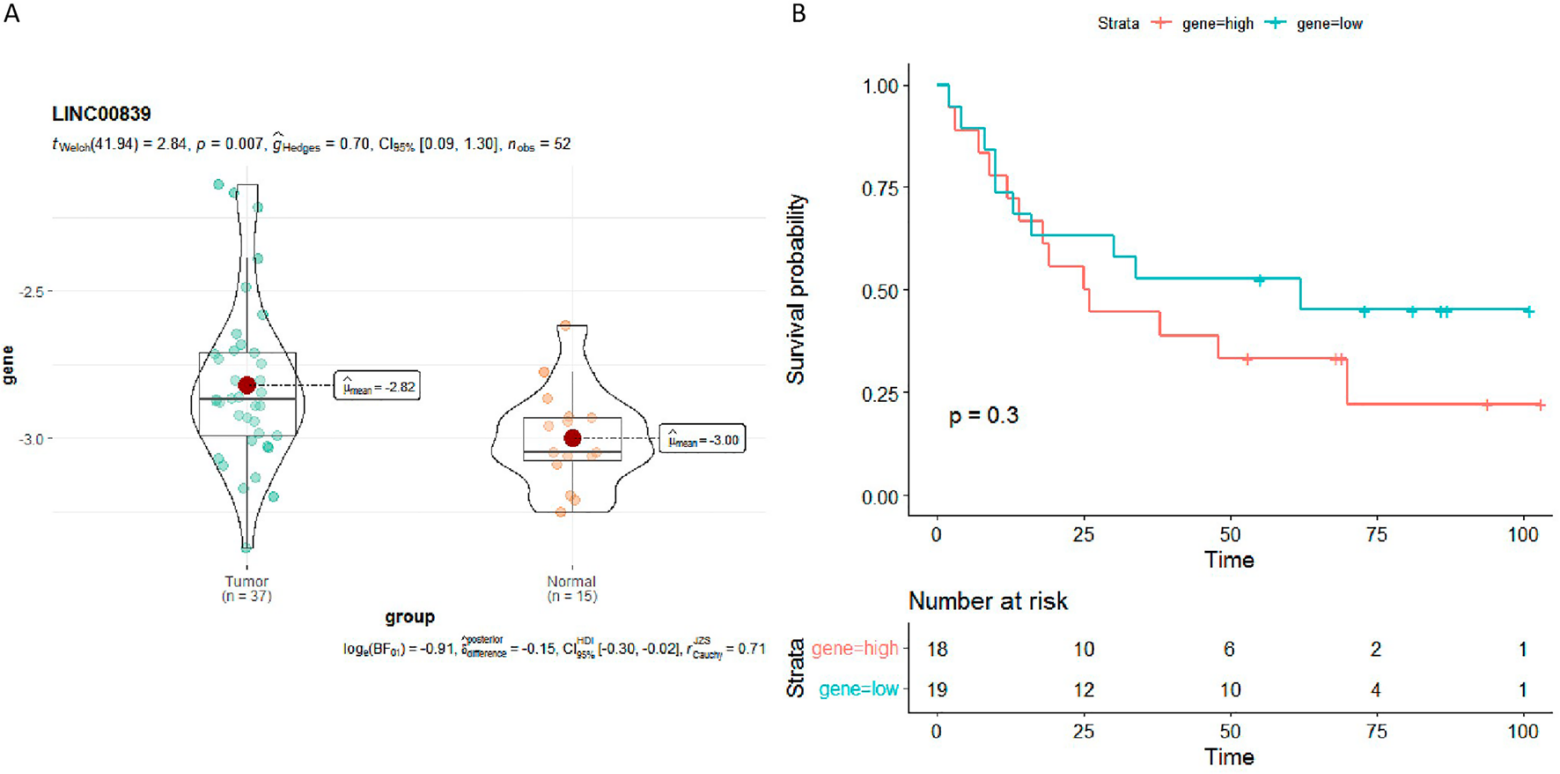
LINC00839 is up-regulated in HCC tissues and correlates with poor OS. (**A**) The LINC00839 expression levels in liver cancer tissue samples (n = 37) and adjacent nontumoral liver tissue samples (n = 15). (**B**) The correlation between LINC00839 levels and patient OS.

The LINC00839 expression levels were further determined in 64 HCC tissue samples and 26 adjacent nontumoral liver tissue samples using qPCR. Unfortunately, LINC00839 expression data were obtained from only 37 HCC tissue samples and 15 nontumoral liver tissue samples. LINC00839 levels were substantially up-regulated in HCC (Fig. 7A). Furthermore, the correlations of LINC00839 levels with clinicopathological features of patients were investigated in the 37 HCC cases. The up-regulation of LINC00839 was positively associated with poor OS (Fig. 7B). The correlations of LINC00839 levels with other clinicopathological features, such as tumor size, number of lesions, and pathological grade were shown in Supplementary Fig. 3.

## Discussion

Approximately, 50–60% of patients with HCC are estimated to be exposed to systemic therapies in their lifespan^2^. Systemic therapies based on immunecheckpoint inhibitor (ICI) were reported to increase overall survival and the quality of life of patients in the past 5 years^28^, underscoring the key role of the tumor microenvironment in the progression of cancer. However, only 15–20% of responders were provided substantial clinical benefits^28^. Understanding the interaction between cancer cells and their microenvironment will be crucial for developing new therapies and identifying biomarkers.

The median oxygen partial pressure (pO_2_) in liver tumors is 6 mm Hg compared with 30 mm Hg in normal liver tissues, revealing that liver tumor oxygenation is heterogeneous and severely compromised^29^. Hypoxia, one of the aberrant physical properties of the tumor microenvironment, can cause broad changes in the epigenome, which are beneficial to the phenotypic selection of hallmark capabilities, including the invasive growth capability of cancer cells^30^. Even so, in preclinical studies, most of cancer cells are cultured in a relatively high ambient atmospheric oxygen concentration (approximately 21% oxygen concentration), which is usually presumed as normoxia, while reality tumors in vivo are usually in a hypoxic state (1–2% oxygen concentration)^31^. Recent studies demonstrated that in different oxygen concentration environments, the physiological state and the sensitivity to drugs of cancer cells were different^32^. Therefore, evaluating cancer cells under hypoxia could more closely recapitulate their physiopathologic status in vivo microenvironment. Our results provided evidence that the malignant phenotypes were significantly enhanced in liver cancer cells under hypoxia, including proliferation, migration, and invasion. Besides, no significant change in cell apoptosis rate was occurred under hypoxia for 24 h, which was consistent with several previous studies^33,34^, suggesting that there was hypoxia-adaptive response in liver cancer cell to attenuate apoptosis. For example, it was demonstrated that HCC cells survived under hypoxia by modulation of mitochondrial dynamics through activation of mitochondrial fission and mitophagy^33^. Nevertheless, another study found that 10% oxygen concentration seemed to provide optimum conditions for long term growth in culture for human cell lines, while oxygen may be inhibitory for growth if its steady-state concentration is below or above optimal, which indicates the possibility that cumulative damage arising from oxygen and products of oxidative metabolism may limit the growth of cells^35^. In fact, even small abnormalities in oxygen concentration can result in altered cellular function^36^. The normoxic condition used in this study was not entirely consistent with the oxygen concentration in vivo, which may result in some results that were not sufficiently objective. Therefore, oxygen levels should be taken into account in genetic studies in vitro, which is likely to be critical to clearly defining and comparing what occurs in whole tissues or tumors.

Recent largescale transcriptome sequencing approaches have identified thousands of lncRNAs that are differentially transcribed between normal tissues and tumors arising from the same organ^12^. It is now recognized that lncRNAs are exquisitely regulated, and can identify clinically relevant cancer subtypes, predict tumor behavior and disease prognosis^37^. Multiple therapeutic strategies have been developed to target lncRNAs. For instance, antisense oligonucleotides (ASOs) are under active investigation to be exploited as RNA inhibitors to treat various diseases^38^. The development of RNA targeting therapeutics provides tremendous opportunities to modulate lncRNAs for anti-cancer purposes. Thus, further exploring the role of lncRNA in the occurrence and development of liver cancer contributes to develop ideal biomarkers for cancer diagnosis and potential drug targets. In this study, we screened two differently expressed lncRNAs with prognostic potential in TCGA database and focused our investigations on the role and potential mechanism of LINC00839 in liver cancer progression and metastasis under hypoxia. Our data revealed that LINC00839 was expressed at a higher level in liver cancer, and increased LINC00839 indicated poor prognosis, suggesting its tumor-promoting effect. LINC00839 was reported to be dysregulated in many kinds of cancer, including HCC^39–42^. However, whether LINC00839 is involved in the hypoxic microenvironment of HCC remains largely elusive. The co-expression analysis revealed that LINC00839 expression levels exhibited a positive correlation with HIF1A expression levels and remarkably, LINC00839 further overexpression was also observed in liver cancer cells cultured under hypoxia. HIF-1 is the most crucial transcription factor under hypoxic stress and binds to the hypoxia response elements (HREs) within the promoter regions of target genes to coordinate cellular transcriptional response^43^. The hypoxia-responsive lncRNAs often act as direct or indirect effectors of HIF-transcriptional cascade, and play pivotal roles in regulating hypoxic gene expression at chromatic, transcriptional, and post-transcriptional levels^44^. The upstream regulator of LINC00839 is largely unknown. This remains the subject of further investigations.

Functionally, this study demonstrated that LINC00839 promoted the proliferation, migration, and invasion of liver cancer cells under hypoxia, suggesting that LINC00839 may be a novel functional regulator of hypoxia-induced signaling. The key roles of lncRNA in gene regulation are linked with their specific subcellular localizations. The lncRNAs localized in the nucleus can modulate chromatin function and regulate the assembly and function of membraneless nuclear bodies, while localized in the cytoplasm, lncRNAs mostly regulate gene expression at the post-transcriptional level, and specific organelle-localized lncRNAs can participate in organelle function and metabolic regulation, such as mitochondrial oxidation and homeostasis^45^. We observed that LINC00839 was localized in both the nucleus and cytoplasm, indicating its functional mechanisms are diverse. The interactions between lncRNAs and binding proteins are central to determine lncRNA functional effects. Therefore, we identified the binding proteins of LINC00839 and performed GO and KEGG pathway enrichment analysis. The proteins were found to be primarily associated with the metabolism and RNA transport. The capability to reprogram cellular metabolism in order to most effectively support neoplastic proliferation now is considered a core hallmark of cancer^30^. LINC00839 responded to hypoxia and may function as a regulator of reprogramming energy metabolism. So the exact function of LINC00839 in liver cancer should be investigated further.

FMNL2 has been disclosed to be required for migration and invasion by transformed cells and strongly implicated in driving tumorigenesis and metastasis of specific tumors^46–48^. Bioinformatic analysis showed that FMNL2 was up-regulated in liver cancer and correlated with patient unfavorable survival..Our data revealed FMNL2 may be a functional partner of LINC00839. LINC00839 could pull down FMNL2, and overexpression of LINC00839 accumulated FMNL2. These experimental results indicated that LINC00839 may promote liver cancer progression by up-regulating FMNL2 expression. However, RIP assay demonstrated that anti-FMNL2 antibody did not enrich LINC00839. Previous studies have shown that FMNL2 binds directly many actin bundling proteins, such as fascin and cortactin, then facilitates cancer cell migration^21,49^. We speculate that FMNL2 exists in the protein complexes pulled down by LINC00839 with other proteins participated in binding of LINC00839 and FMNL2. Further research is warranted to illustrate a more detail mechanism.

We also found correlations of LINC00839 levels with clinicopathological features of patients. The higher LINC00839 expression was positively associated with worse prognosis and bigger tumor size. However, a study with a larger cohort is needed to verify the clinical significance of elevated LINC00839 expression. Additionally, this study lacks of in vivo experiments, which ought to be supplemented for a better understanding of the effects of LINC00839 on liver cancer.

In conclusion, our study reported that LINC00839 was significantly up-regulated in liver cancer under hypoxia. LINC00839 promoted liver cancer cell proliferation, migration, and invasion under hypoxia. More importantly, LINC00839 may function as a regulator of reprogramming energy metabolism and can up-regulate the protein expression level of FMNL2. Hence, LINC00839 is implicated as a potential target for anticancer therapy.

## Supplementary Information

Supplementary Information 1.

Supplementary Information 2.

Supplementary Information 3.

Supplementary Information 4.

Supplementary Legends.

## Data Availability

The data generated or analysed during this study are included in this published article and its supplementary information files, and the mass spectrometry proteomics data have been deposited to the ProteomeXchange Consortium via the PRIDE^50^ partner repository with the dataset identifier PXD034526.
