# Supplementary figures and images for: LINC00839 promotes malignancy of liver cancer via binding FMNL2 under hypoxia
Source: Sci Rep. 2022 Nov 5;12:18757. doi: 10.1038/s41598-022-16972-z (PMC9637198; doi:10.1038/s41598-022-16972-z)

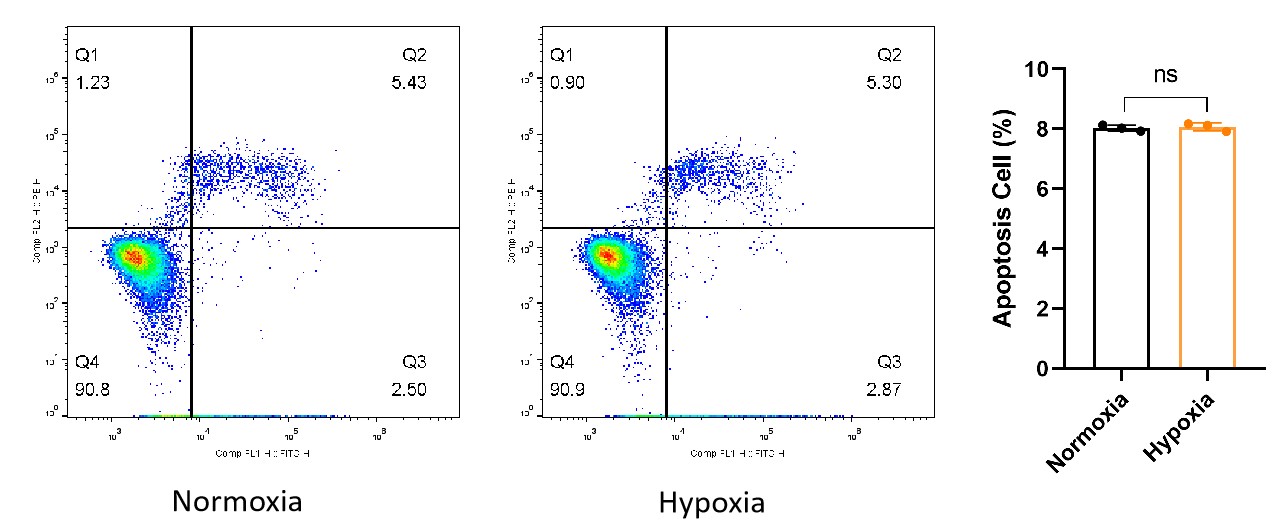

Supplement: Supplementary file 1 — Supplementary Information 1. [file 41598_2022_16972_MOESM1_ESM.jpg]

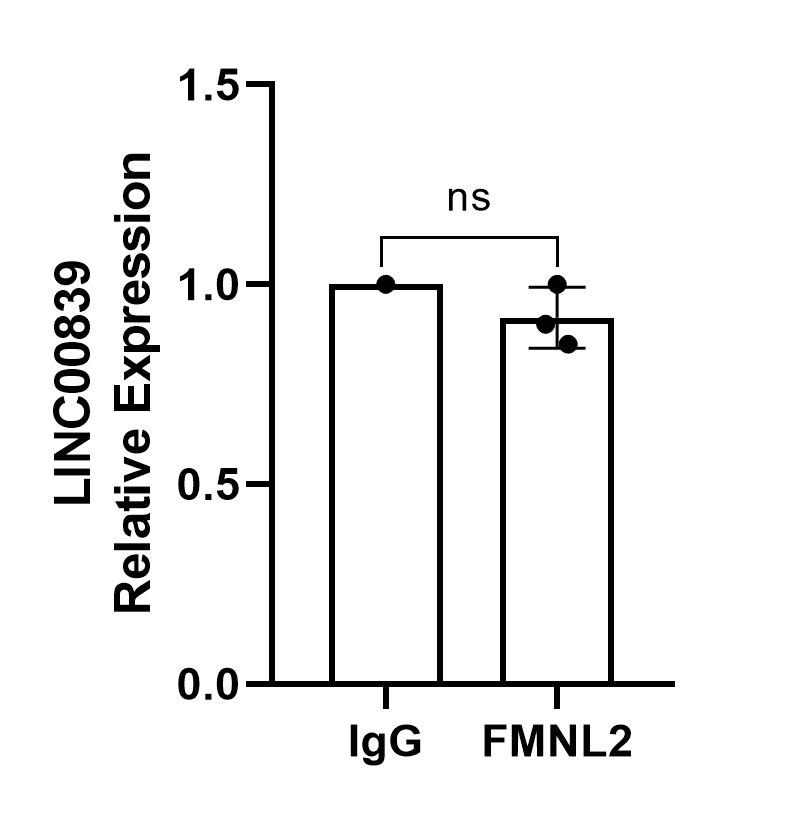

Supplement: Supplementary file 2 — Supplementary Information 2. [file 41598_2022_16972_MOESM2_ESM.jpg]

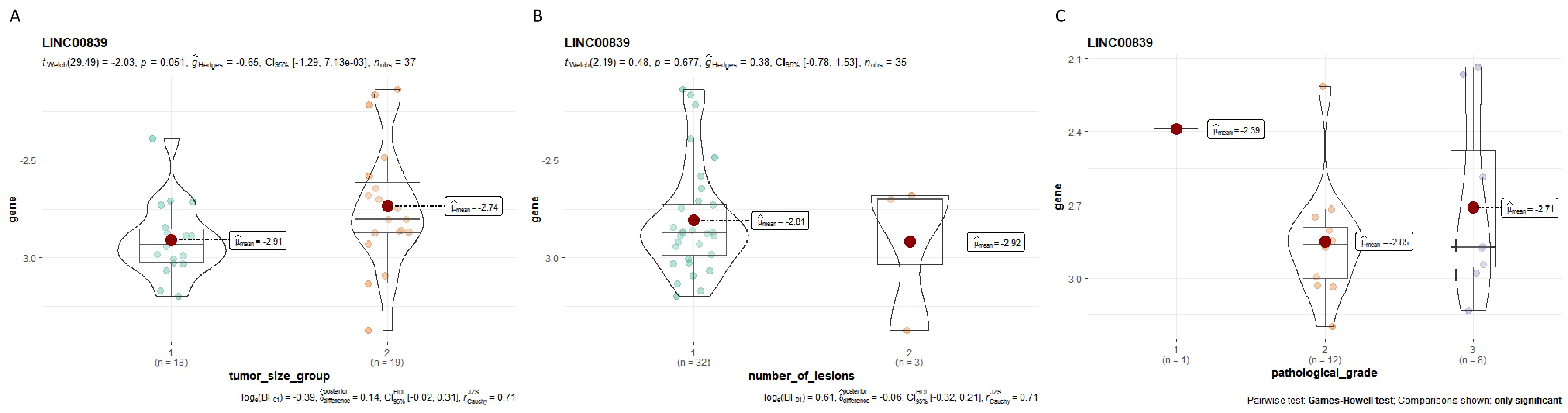

Supplement: Supplementary file 3 — Supplementary Information 3. [file 41598_2022_16972_MOESM3_ESM.jpg]

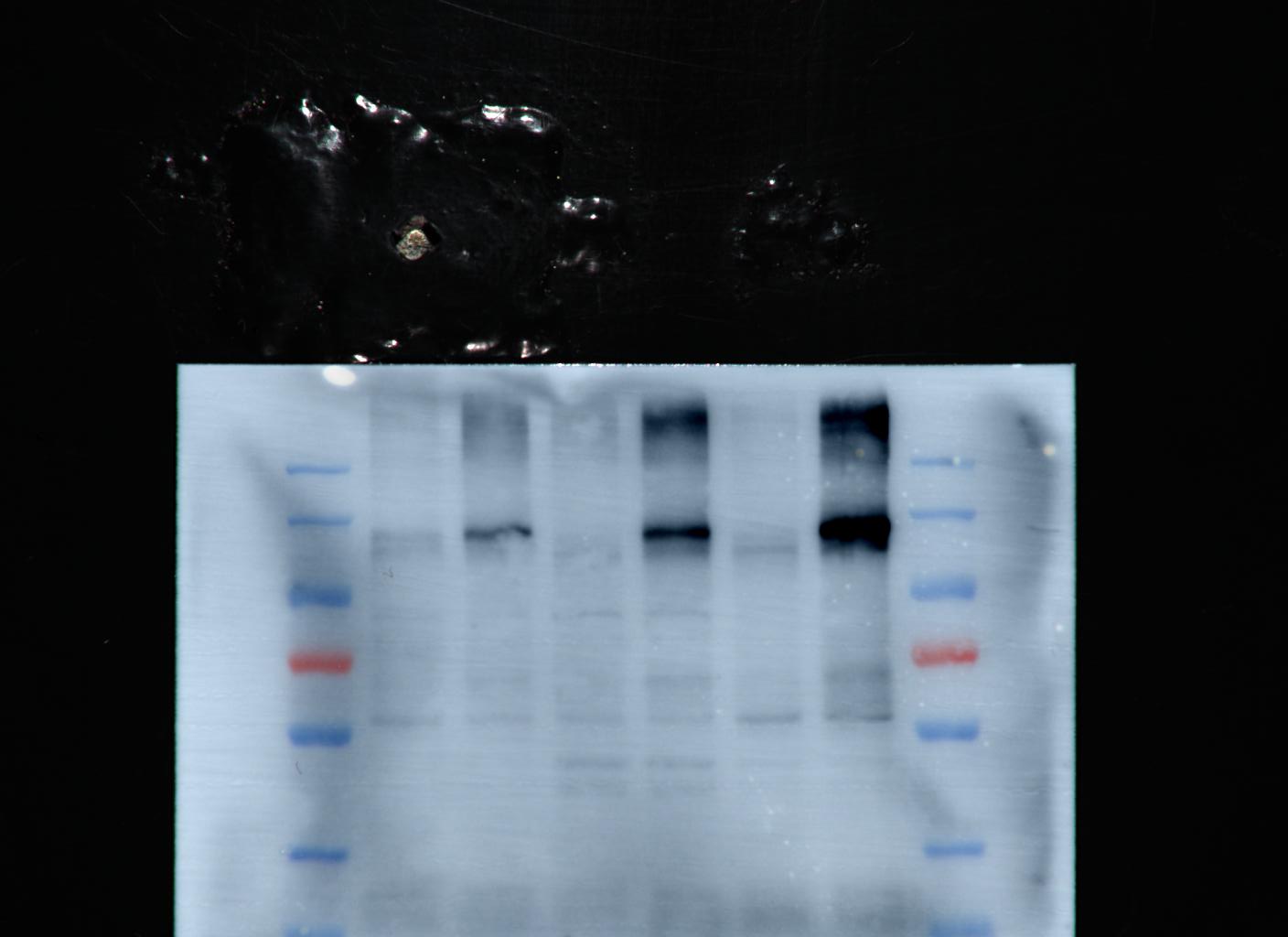

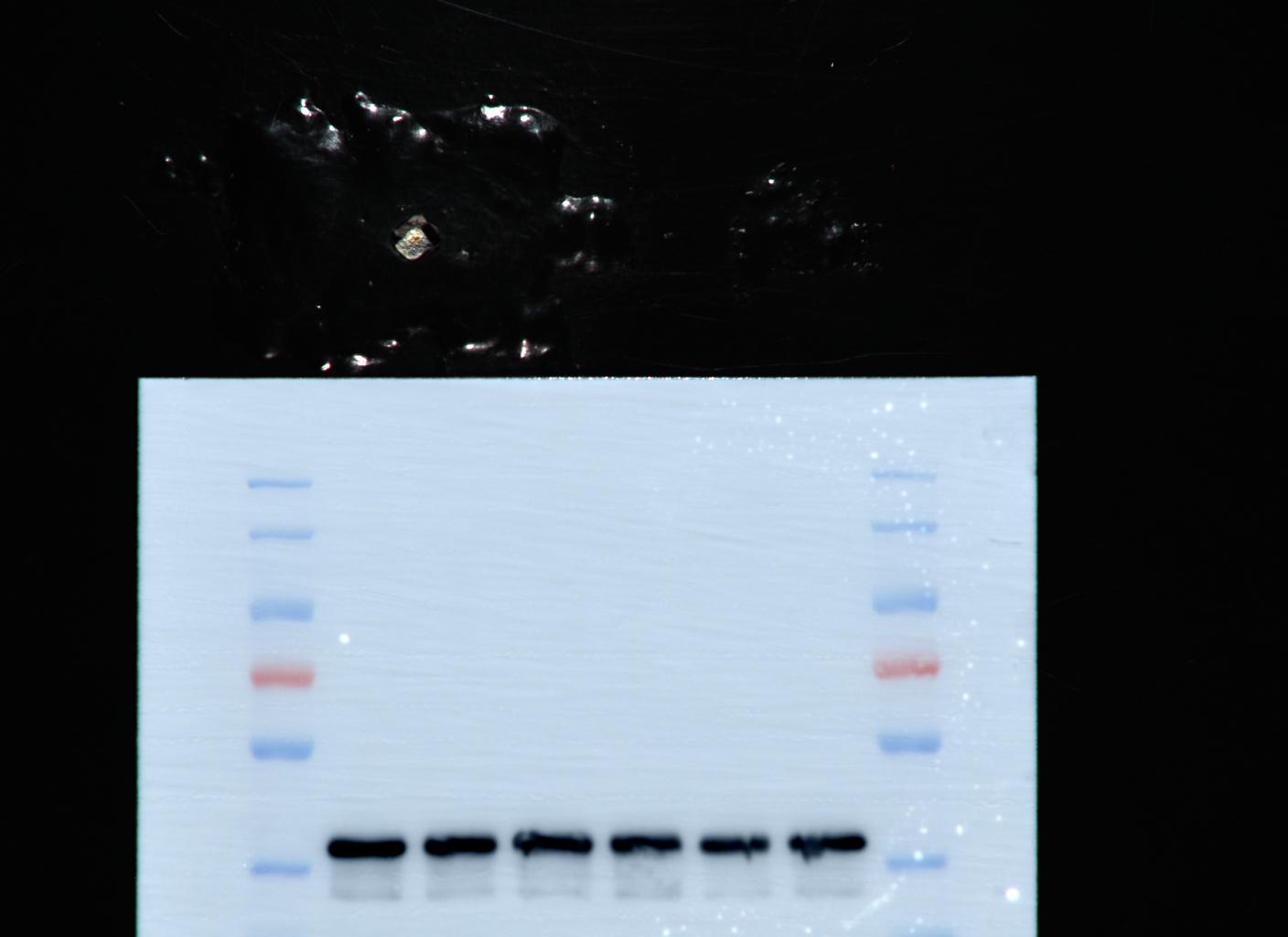


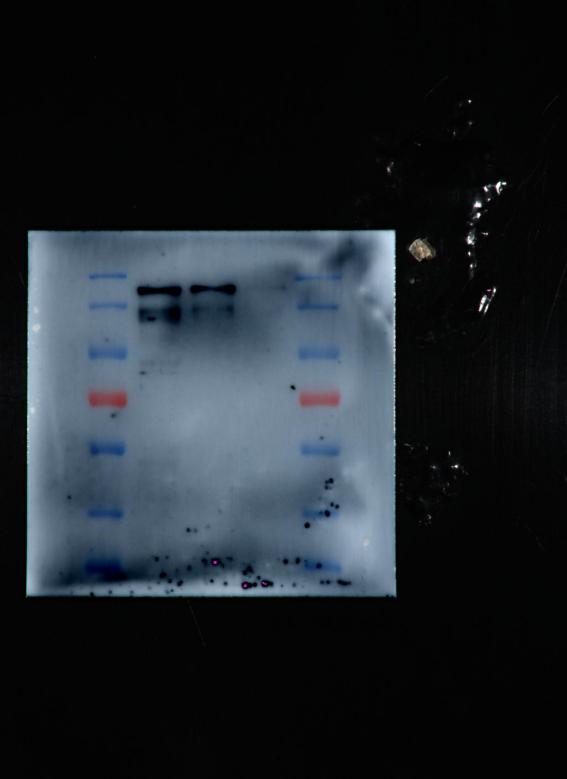

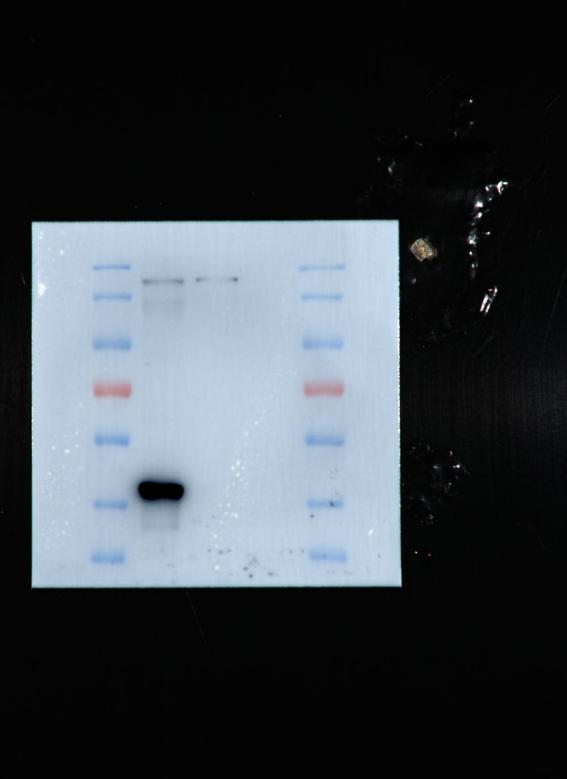

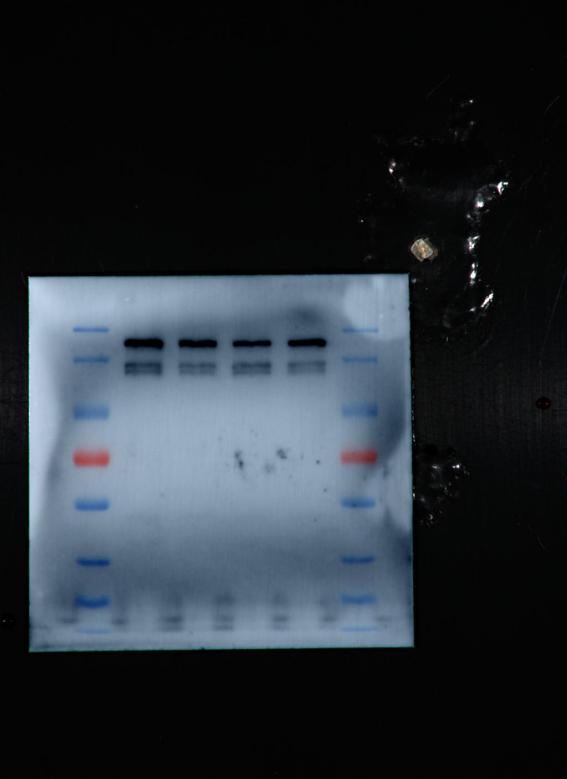

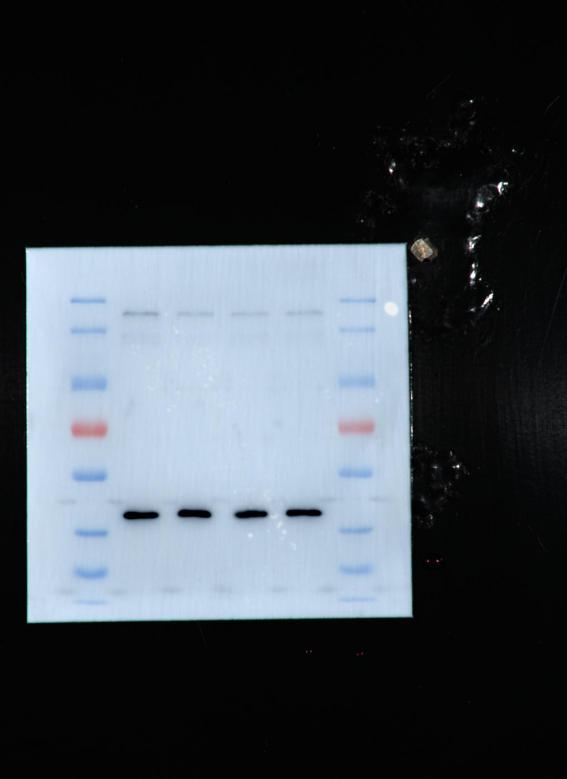

Supplement: Supplementary file 4 — Supplementary Information 4. [file 41598_2022_16972_MOESM4_ESM.docx]
