# Supplementary material for: LINC00839 promotes malignancy of liver cancer via binding FMNL2 under hypoxia
Source: Sci Rep. 2022 Nov 5;12:18757. doi: 10.1038/s41598-022-16972-z (PMC9637198; doi:10.1038/s41598-022-16972-z)
Supplement: Supplementary file 5 — Supplementary Legends. [file 41598_2022_16972_MOESM5_ESM.docx]

**Supplementary Figure 1.** Hypoxia did not affect liver cancer cell apoptosis.

**Supplementary Figure 2.** The RIP assay showed that anti-FMNL2 antibody couldn’t enrich LINC00839, compared with IgG group.

**Supplementary Figure 3.** The correlations of LINC00839 levels with clinicopathological features. (**A**) The correlations of LINC00839 levels with tumor size. Group 1 means ≤ 5 cm; Group 2 means ＞ 5 cm. (**B**) The correlations of LINC00839 levels with number of lesions. (**C**) The correlations of LINC00839 levels with pathological grade.

**Supplementary File 1.** The original WB results with markers.
